# Within- and across-network alterations of the sensorimotor network in Parkinson’s disease

**DOI:** 10.1007/s00234-021-02731-w

**Published:** 2021-05-21

**Authors:** Julian Caspers, Christian Rubbert, Simon B. Eickhoff, Felix Hoffstaedter, Martin Südmeyer, Christian J. Hartmann, Benjamin Sigl, Nikolas Teichert, Joel Aissa, Bernd Turowski, Alfons Schnitzler, Christian Mathys

**Affiliations:** 1 Department of Diagnostic and Interventional Radiology, University Düsseldorf, Moorenstr. 5, D-40225 Düsseldorf, Germany; 2 Institute of Neuroscience and Medicine (INM-1, INM-7), Research Centre Jülich, 52425 Jülich, Germany; 3 Institute of Systems Neuroscience, Heinrich Heine University, 40225 Düsseldorf, Germany; 4 Department of Neurology, Ernst-von-Bergmann Klinikum, 14467 Potsdam, Germany; 5 Department of Neurology, Center for Movement Disorders and Neuromodulation, Heinrich Heine University, 40225 Düsseldorf, Germany; 6 Institute of Clinical Neuroscience and Medical Psychology, Heinrich Heine University, 40225 Düsseldorf, Germany; 7 Evangelisches Krankenhaus, Institute of Radiology and Neuroradiology, Carl von Ossietzky University Oldenburg, D-26122 Oldenburg, Germany; 8 Research Centre Neurosensory Science, Carl von Ossietzky University Oldenburg, D-26129 Oldenburg, Germany

**Keywords:** Resting-state fMRI, Functional connectivity, Parkinson’s disease, Sensorimotor integration, Dopaminergic therapy

## Abstract

**Purpose:**

Parkinson’s disease (PD) is primarily defined by motor symptoms and is associated with alterations of sensorimotor areas. Evidence for network changes of the sensorimotor network (SMN) in PD is inconsistent and a systematic evaluation of SMN in PD yet missing. We investigate functional connectivity changes of the SMN in PD, both, within the network, and to other large-scale connectivity networks.

**Methods:**

Resting-state fMRI was assessed in 38 PD patients under long-term dopaminergic treatment and 43 matched healthy controls (HC). Independent component analysis (ICA) into 20 components was conducted and the SMN was identified within the resulting networks. Functional connectivity within the SMN was analyzed using a dual regression approach. Connectivity between the SMN and the other networks from group ICA was investigated with FSLNets. We investigated for functional connectivity changes between patients and controls as well as between medication states (OFF vs. ON) in PD and for correlations with clinical parameters.

**Results:**

There was decreased functional connectivity within the SMN in left inferior parietal and primary somatosensory cortex in PD OFF. Across networks, connectivity between SMN and two motor networks as well as two visual networks was diminished in PD OFF. All connectivity decreases partially normalized in PD ON.

**Conclusion:**

PD is accompanied by functional connectivity losses of the SMN, both, within the network and in interaction to other networks. The connectivity changes in short- and long-range connections are probably related to impaired sensory integration for motor function in PD. SMN decoupling can be partially compensated by dopaminergic therapy.

**Supplementary Information:**

The online version contains supplementary material available at 10.1007/s00234-021-02731-w.

## Introduction

Parkinson’s disease (PD) is one of the most common neurodegenerative diseases and is primarily defined by its motor symptoms comprising the classical clinical triad of bradykinesia, rigidity, and tremor. Loss of dopaminergic neurons within the substantia nigra pars compacta is regarded as the primary pathological hallmark of the disease and the resulting destabilization of basal ganglia circuits following nigrostriatal dopamine depletion is thought to be the main cause of deficits in motor control in PD. However, pathophysiological changes in PD clearly exceed the level of the basal ganglia yielding extensive alterations of cortico-striatal and cortico-cortical connections as a consequence of complex neurochemical imbalances [1, 2].

In recent years, advanced neuroimaging techniques could strongly contribute to capture these changes in functional connections associated with PD and promoted a rapid progress in the deeper understanding of the disease [3]. One of the most strongly emerging fields in functional neuroimaging in the last years is resting-state functional magnetic resonance imaging (fMRI), which facilitates the analysis of functional connections within and between brain networks without the need for subjects to perform a specific task. This rather simple acquisition makes this technique feasible for a larger patient group compared to classical (task-based) fMRI due to lower requirements on patient cooperation. Resting-state functional connectivity (FC) has been widely used to investigate PD and other neurological and psychiatric diseases and is currently considered to have great potential as a neuroimaging biomarker to enhance diagnosis and disease monitoring in these disorders [4, 5].

Research on FC has highlighted that the brain is intrinsically organized into distinct large-scale connectivity networks, which facilitate human brain function by their dynamic interplay [6, 7]. Each network consists of several remote brain regions that show common and highly synchronized neuronal activity, and is associated with specific mental operations. Among the most commonly and consistently reported intrinsic connectivity networks are the default mode network [8], the fronto-parietal executive control networks [9], and the sensorimotor network (SMN) [10, 11].

**Fig. 1:**
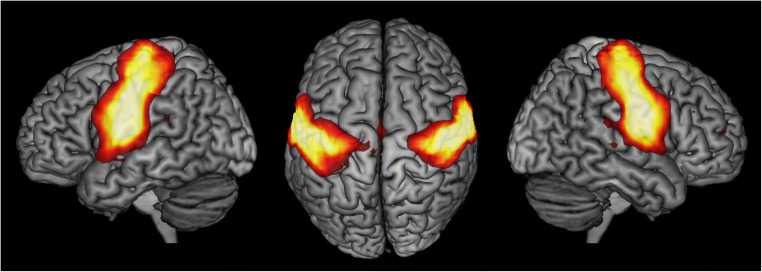
The sensorimotor network (SMN) projected onto a 3D rendering of the MNI single-subject reference brain. Left, top, and right views are shown

The SMN integrates primary sensorimotor, premotor and supplementary motor areas (SMA) to facilitate voluntary movements (Fig. 1). Its intrinsic activity at rest resembles activation seen in motor tasks [11]. Neuroimaging studies have repeatedly shown disease-related alterations in sensorimotor areas in PD [12], e.g., hypoactivation of the SMA and primary motor cortex in simple finger movement tasks [13] or decreased FC within motor areas [14]. However, reported results are not consistent across studies and mostly base on selected patient samples, e.g., only drug-naïve patients. A systematic analysis of the SMN in PD patients of different stages on a network basis and its relation to dopaminergic treatment is yet missing.

The current study investigates the within- and across-network connectivity of the SMN in PD patients under long-term dopaminergic treatment based on independent component analysis (ICA) of resting-state fMRI and tests for disease-related connectivity changes as well as the effect of dopaminergic medication.

## Material and methods

### Sample

Thirty-eight patients from the Center for Movement Disorders and Neuromodulation at the University Hospital Düsseldorf diagnosed with idiopathic PD and 43 healthy controls (HC) without any record of neurological or psychiatric disorders were included in the current analysis. PD patients and HC were matched for age, gender, and within-scanner head movement. Subjects were selected from an existing pool of 82 PD patients and 78 HC by determining the largest subsample from all subjects with complete and sufficient imaging data and no exclusion criteria, so that age, gender, and motion parameters from EPI motion correction did not differ significantly between patients and HC (two-sample t-tests for age and motion parameters, x^2^-test for gender, all p > 0.1). All patients were examined by an experienced movement disorders specialist. Diagnosis of idiopathic PD was based on the UK Parkinson’s Disease Society Brain Bank Clinical Diagnostic Criteria [15] following thorough diagnostic workup including neurological examination, clinical history and family anamnesis, neuropsychiatric testing, neuroimaging, and levodopa testing in all patients. The patient sample comprised all stages of the disease on the Hoehn & Yahr scale. All patients were under long-term dopaminergic treatment with individual drug regimens optimized for their needs, including levodopa, catechol-O-methyl-transferase inhibitors, dopamine agonists, and further symptomatic drugs. The levodopa equivalent dose (LED) of each patient was calculated according to Tomlinson et al. [16]. Patients underwent resting-state fMRI while under their regular dopaminergic medication (ON), and, additionally, after at least 12-h overnight withdrawal of all dopaminergic drugs (OFF). Severity of motor symptoms in the ON and OFF states was assessed by the Unified Parkinson’s Disease Rating Scale Part III (UPDRS-III).

Exclusion criteria were non-idiopathic Parkinsonism, severe dementia, major depression, and ineligibility for MRI. Subjects with substantial head movement in their fMRI scan (mean relative RMS displacement > 0.4 mm) were excluded prior to the matching procedure.

Demographic and clinical characteristics of the sample are given in Table 1.

**Table 1:** Sample characteristics (*MDRS score was available in 37 of 38 patients; **MoCA score was available in 31 of 38 patients)

	Healthy controls	PD patients
Number	43	38
Age (years): mean ± sd	59.7 ± 8.9	61.3 ± 9.5
Sex: female/male	19 (44%)/24 (56%)	15 (39%)/23 (61%)
Disease duration (years): mean ± sd		9.9 ± 5.0
Hoehn & Yahr stage: median (IQR)		3 (2–3)
UPDRS-III (OFF): median (IQR)		34 (26.25–40.5)
UPDRS-III (ON): median (IQR)		17.5 (12.5–25)
LED: mean ± sd		1078.6 ± 342.2
Mattis Dementia Rating Scale (MDRS)*: median (IQR)		139 (137–142)
Montreal Cognitive Assessment (MoCA)**: median (IQR)		27 (22.5–28)
Symptom lateralization: right/left		14 (37%)/24 (63%)
Motor type: akinetic rigid/tremor dominant/mixed type		13 (34%)/5 (13%)/20 (53%)

### MRI acquisition and preprocessing

Resting-state fMRI was acquired using an echo-planar imaging (EPI) sequence covering the whole brain over a time period of 11 min on a 3 T Siemens Trio (Siemens, Erlangen, Germany) to obtain blood oxygen level-dependent (BOLD) time series (300 time points, TR = 2.2 s, TE = 30 ms, flip angle = 90°, FoV = 200 × 200 mm^2^ axial plane, slices = 36, voxel size = 3.1 × 3.1 × 3.1 mm^3^). Patients were instructed to keep their eyes closed and let their mind flow without thinking at anything particular. T1-weighted structural MRI scans were acquired using a three-dimensional magnetization prepared rapid gradient-echo sequence (MPRAGE, TR = 2.3 s, TE = 2.96 ms, TI = 900 ms, flip angle = 8°, FoV = 240 × 256 mm^2^ sagittal plane, slices = 192, voxel size = 1 × 1 × 1 mm^3^).

Preprocessing and data analysis of MRI data was conducted with the Oxford Centre for Functional MRI of the Brain (FMRIB) Software Library (FSL) version 5.0 [17].

The first five images of fMRI time series were discarded to account for magnetic saturation effects. EPI volumes were then motion corrected and slice timing correction was conducted. The six rigid-body parameter time series yielded from motion correction were used for later EPI signal denoising, and mean relative RMS displacement was used for group matching (see above). Motion-corrected fMRI volumes as well as structural MRIs were then brain extracted with FMRIB’s Brain Extraction Tool [18]. EPIs were spatially smoothed with a 5-mm full width at half maximum Gaussian kernel, intensity normalized across time series, and high-pass filtered with a cutoff of 150 s. Then, automated signal denoising of fMRI data was conducted applying the FMRIB ICA-based Xnoisifier (FIX) [19, 20]. Finally, EPIs were linearly co-registered to their 3D structural image, and subsequently spatially normalized to MNI152 standard reference space by applying the deformations yielded from linear and non-linear registration of the structural image to MNI152.

### Group-level independent component analysis

**Fig. 2:**
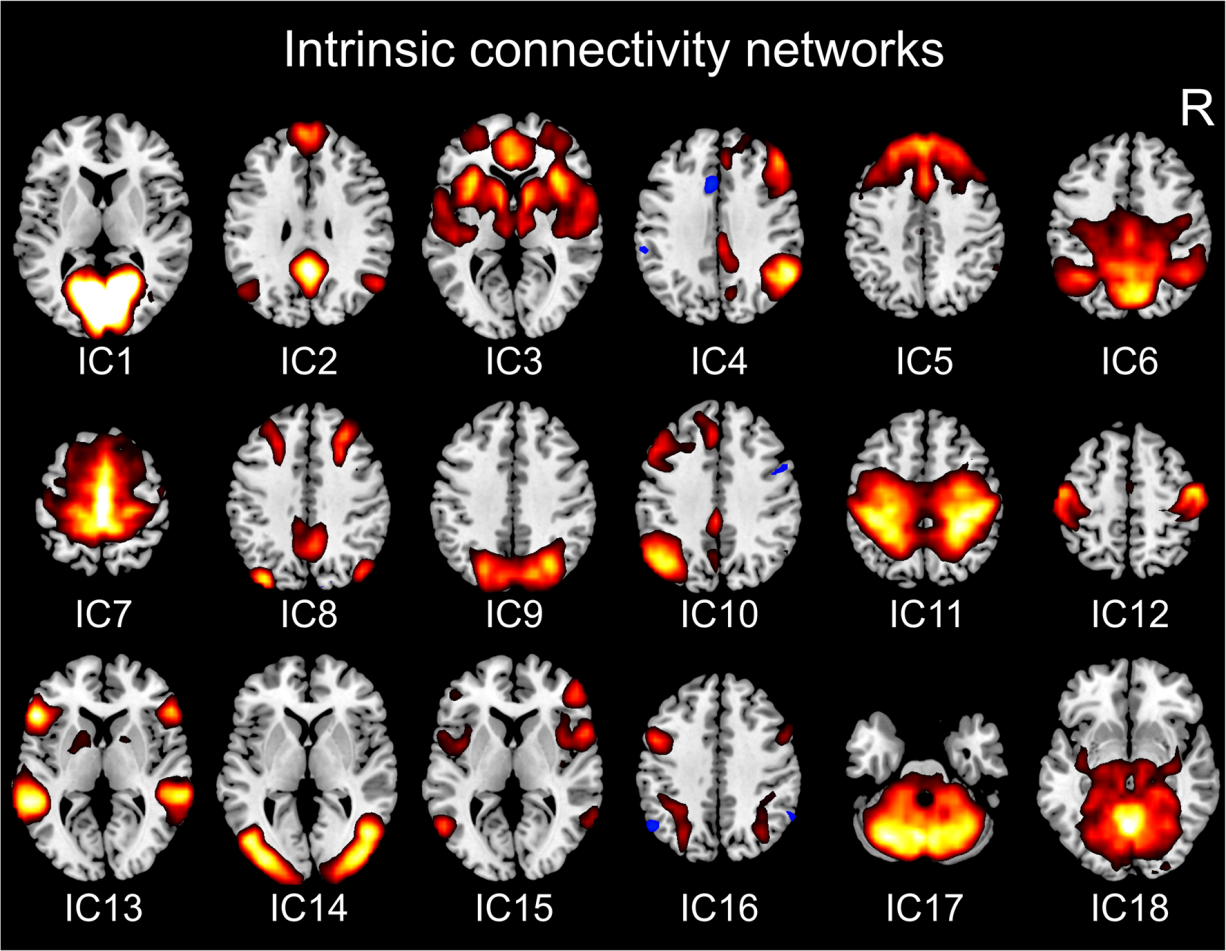
Intrinsic connectivity networks from group-level independent components. Representative axial slices for each independent component (IC) are shown

Intrinsic connectivity networks were derived from probabilistic ICA of the fMRI data of healthy controls using FMRIB’s Multivariate Exploratory Linear Optimized Decomposition into Independent Components (MELODIC) [21]. For this, the respective preprocessed EPI volumes were temporally concatenated to a single 4D volume, which was then split into 20 spatially independent components applying variance normalization (Fig. 2). Resulting spatial components were checked for residual artificial components based on their spatial distribution and time series power spectrum and were then assigned to known intrinsic connectivity networks [22–24]. Based on these criteria, two components (IC19, IC20, Suppl. Fig. 1) were discarded for further analysis as noise components. The SMN could be sufficiently identified within the remaining 18 components (IC12, Fig. 2).

### Intra-network functional connectivity analysis

FC alterations between PD patients and HC within the SMN were evaluated using a dual regression approach [25, 26]. In the first step, subject-specific SMN time series were generated for each participant by using the SMN spatial map from group ICA as a spatial regressor in each subject’s preprocessed 4D EPI. Resulting time series were variance normalized. In the second step, the time series obtained from the first step of dual regression were used as temporal regressors in the same 4D datasets to estimate subject-specific spatial representations of the SMN. Then, voxel-wise group comparisons and correlation analyses were performed on the resulting subject-specific maps in separate general linear models (GLMs) by applying permutation testing (5000 permutations). Voxel-wise analyses were restricted to all brain voxels present in all subjects included for the specific comparison using a binary mask. We analyzed for group differences in SMN FC, between HC and PD patients in both medical states (HC vs. PD OFF, HC vs. PD ON) using a two-sample t-test design as well as between the two medical states of patients (PD OFF vs PD ON) with a paired t-test design. Possible correlations of patients’ SMN FC with disease duration, UPDRS-III in medical OFF and ON, absolute UPDRS-III improvement from medical OFF to ON, motor subtype, and LED were analyzed for the scans from both medical conditions in separate GLMs. Results were considered significant at p < 0.05 corrected for multiple comparisons by controlling the family-wise error rate (FWE) and applying threshold-free cluster enhancement (TFCE) [27]. To report correlation coefficients for significant clusters from voxel-wise correlation analyses, post hoc Pearson correlations between the mean functional connectivity of the cluster to SMN and the respective covariate were calculated.

Brain regions resulting from intra-network connectivity analyses were anatomically allocated to probabilistic cytoarchitectonic maps using JuBrain, the Jülich brain Atlas [28], as implemented in the SPM Anatomy Toolbox V.2.0 [29, 30].

### Inter-network functional connectivity analysis

Inter-network FC alterations of the SMN, i.e., connectivity changes between the SMN and other intrinsic connectivity networks, were analyzed using the FSLNets package (https://fsl.fmrib.ox.ac.uk/fsl/fslwiki/FSLNets) in MATLAB (The MathWorks, Natick, MA, USA). For this, the normalized subject-specific time series obtained from the first step of dual regression for the SMN and the 17 remaining components resulting from group ICA were used as input for FSLNets. For all subjects, Pearson correlation coefficients between the time series of the SMN and each of the other 17 networks were calculated and transformed into Fisher’s z-scores. Group comparisons and correlation analyses were performed on these connectivity z-scores using the same GLM designs as for the intra-network analyses (see above) and applying permutation testing (5000 permutations). Resulting inter-network correlations were considered significant at a p < 0.05 threshold, FWE corrected for multiple comparisons.

## Results

### Intra-network functional connectivity analysis

**Fig. 3:**
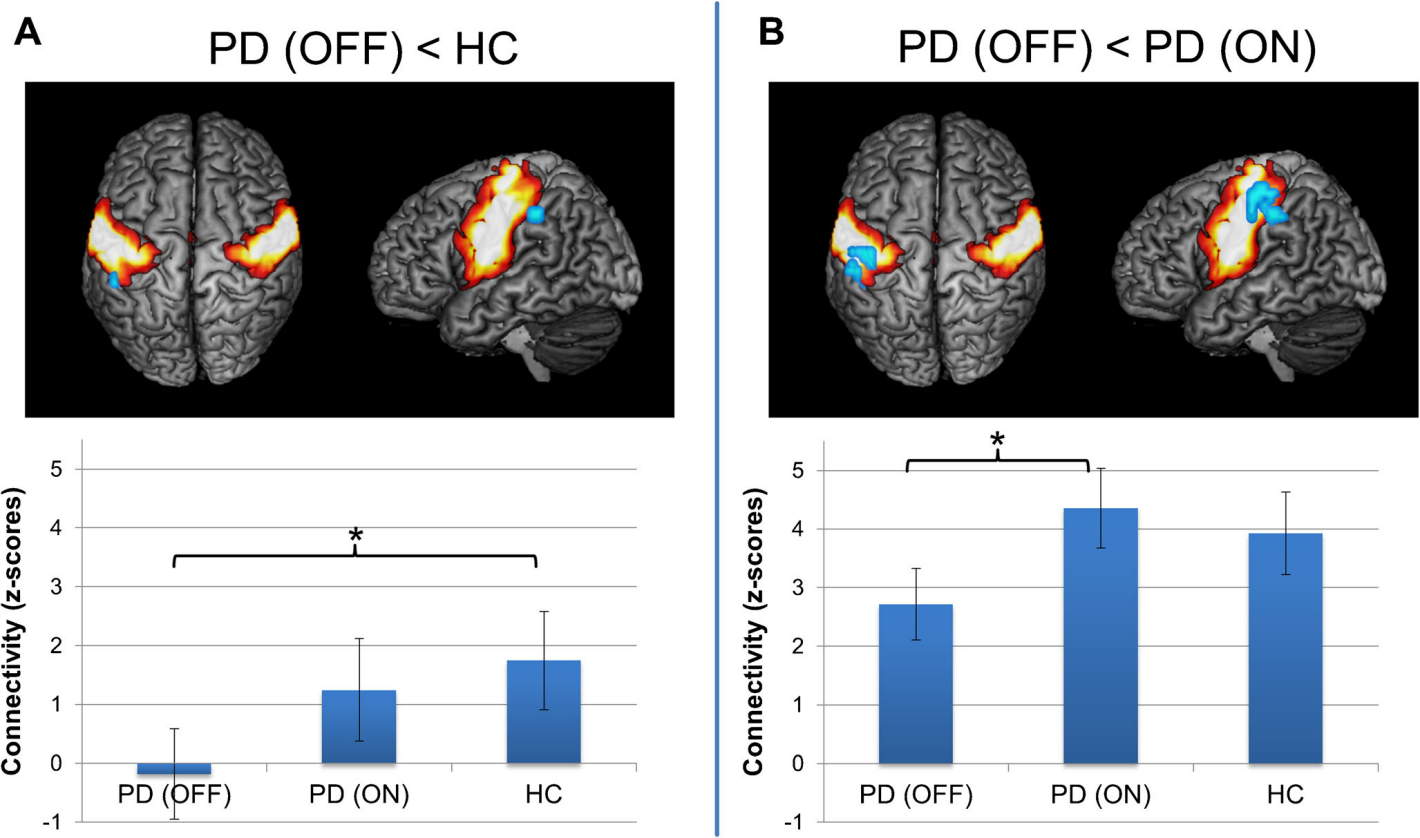
Results from intra-network functional connectivity analysis of the sensorimotor network (SMN) between PD patients in medical OFF and healthy controls (A) and between both medical states in PD patients (B). Significant functional connectivity decreases within the SMN (cold colors) are projected onto the SMN (hot colors) on a 3D rendering of the MNI single-subject reference brain. Top and left lateral views are shown. Bar plots in bottom row indicate functional connectivity (z-scores) of the altered regions across subject groups and medical states. Whiskers indicate standard deviation. Brackets with asterisks indicate significant results between groups (p < 0.05, corrected for multiple-comparison correction using family-wise error correction)

Results for intra-network functional connectivity analysis are given in Table 2. We found significantly reduced FC within the SMN in area PFt [31] of the left inferior parietal lobule (IPL) in PD OFF compared to HC (Fig. 3A). There were no significant connectivity decreases for PD ON compared to HC. When comparing both medical conditions in PD patients, there was decreased FC in left area PFt of the IPL and in area 3b [32] of the left postcentral gyrus at the somatotopic level of the somatosensory hand and face representation in PD OFF compared to PD ON (Fig. 3B). There were no significant connectivity increases for any group comparison.

**Table 2:** Significant results from intra-network functional connectivity group comparison and correlation analyses of the SMN

Contrast/analysis	Anatomical region	Number of voxels	Center of gravity (MNI space)		
			x	Y	z
PD OFF < HC	Left IPL (area PFt)	4	−50	−36	42
PD OFF < PD ON	Left IPL (area PFt)/left postcentral gyrus (area 3b)	38	−47	−25	49
	Left postcentral gyrus (area 3b)	2	−54	−18	42
Correlation: FC(PD OFF)–disease duration	Left parietal operculum (area OP1)	5	−52	−22	13

When quantifying the FC of these altered regions across subjects, mean FC in left area PFt was nearly zero in PD OFF and showed an increase in PD ON to a value lower than HC. For left area 3b, connectivity in PD OFF was likewise decreased compared to HC, but connectivity increase in PD ON even exceeded FC of HC.

We found a significant positive correlation between disease duration and within SMN FC in area OP1 [33] of the left parietal operculum in PD OFF (r = 0.78, p < 0.001). There were no significant correlations with intra-network FC for UPDRS-III, UPDRS-III improvement, motor subtype, or LED.

### Inter-network functional connectivity analysis

**Fig. 4:**
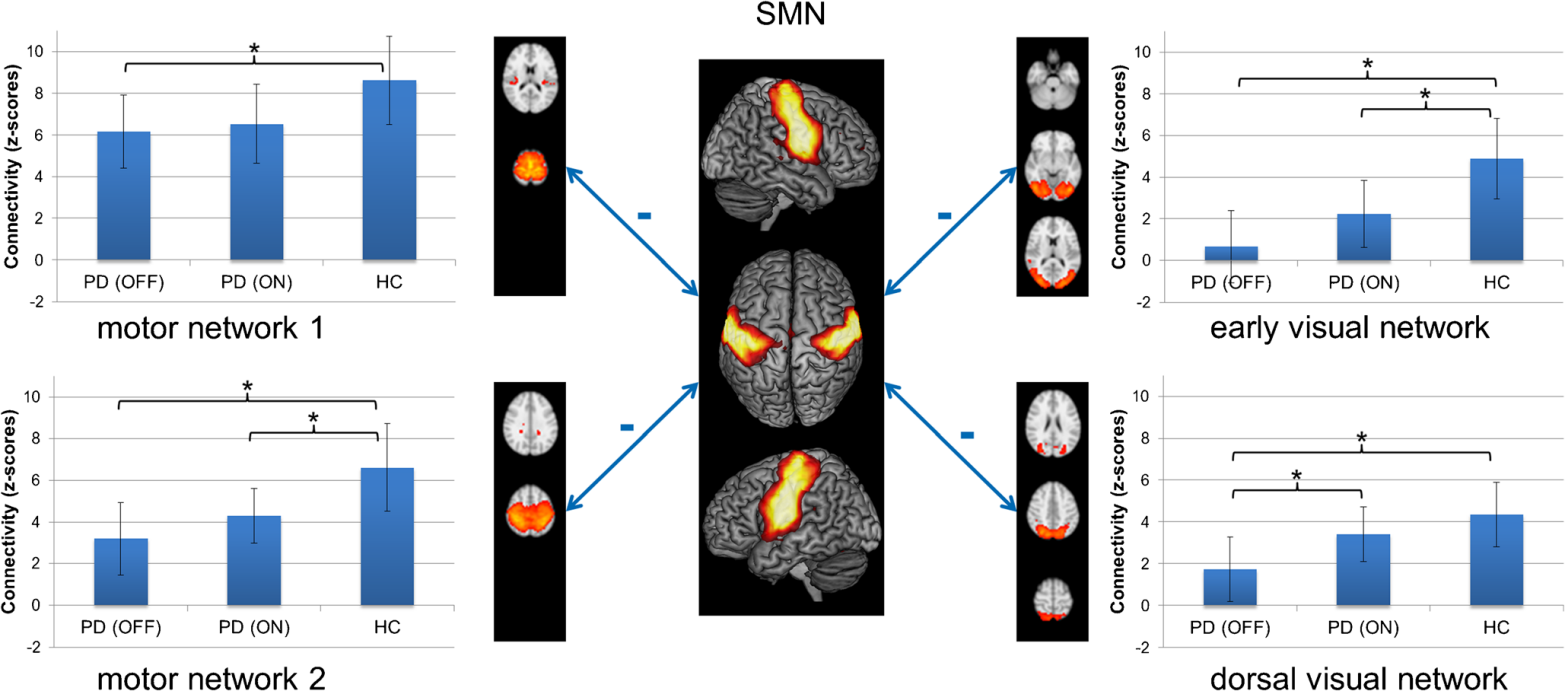
Results from inter-network functional connectivity of the sensorimotor network (SMN). Double-headed arrows with minus indicate significant functional connectivity decreases between the SMN and two motor networks, an early and a dorsal visual network. Representative axial sections of the four altered networks are shown. Bar plots next to the network images indicate functional connectivity (z-scores) between SMN and the respective network across subject groups and medical states. Whiskers indicate standard deviation. Brackets with asterisks indicate significant results between groups (p < 0.05, corrected for multiple-comparison correction using family-wise error correction)

The analysis of inter-network connectivity of the SMN in FSLNets (Fig. 4, Table 3) yielded significantly decreased FC between the SMN and two motor associated networks, an early visual network and a dorsal visual/superior parietal network in PD OFF compared to HC. The first motor network (IC7) was mainly located in the medial precentral and postcentral gyrus as well as posterior medial prefrontal areas, and additionally involved the temporoparietal junction, parietal operculum and posterior insula, and the thalamus, bilaterally. The second motor network (IC11) was located in the precentral and postcentral gyri, partially reaching into the premotor cortex and superior parietal lobule of both sides. The early visual network (IC14) comprised ventral and dorsal visual areas hOC3v, hOC4v [34], hOC3d, hOC4d [35], FG1, and FG2 [36], sparing the primary and secondary visual cortex. The dorsal visual/superior parietal component (IC9) involved the dorsal visual cortex comprising areas hOC3d and hOC4d and the posterior superior parietal lobule covering areas 7P and 7A [37].

**Table 3:** Significant results from inter-network group comparison and correlation analyses. Given p-values are corrected for multiple comparisons using family-wise error correction

Contrast/analysis	Inter-network connection	p-value/r-value
PD OFF < HC	SMN – motor network 1 (IC7)	p = 0.045
	SMN – dorsal visual network (IC9)	p = 0.004
	SMN – motor network 2 (IC11)	p = 0.003
	SMN – early visual network (IC14)	p < 0.001
PD ON < HC	SMN – motor network 2 (IC11)	p = 0.032
	SMN – early visual network (IC14)	p = 0.011
PD OFF < PD ON	SMN – dorsal visual network (IC9)	p = 0.026
Correlation: FC(PD OFF)–disease duration	SMN – motor network 1 (IC7)	p = 0.002 ; r = −0.48
Correlation: FC(PD ON)–disease duration	SMN – basal ganglia/salience network (IC3)	p < 0.001 ; r = −0.66
	SMN – right fronto-parietal network (IC4)	p = 0.001 ; r = −0.50
	SMN – dorsal visual network (IC9)	p = 0.006 ; r = −0.44
	SMN – language network (IC15)	p = 0.004 ; r = −0.46
Correlation: FC(PD ON)–UPDRS-III (OFF)	SMN – motor network 1 (IC7)	p = 0.002, r = −0.49
Correlation: FC(PD ON)–ΔUPDRS-III	SMN – right fronto-parietal network (IC4)	p = 0.005; r = −0.45

Significantly reduced FC of SMN to one motor network (IC11) as well as to the early visual network (IC14) was also found for PD ON compared to HC. Furthermore, there was a significantly lower FC between SMN and the dorsal visual/superior parietal network (IC9) in PD OFF compared to PD ON.

When quantifying the SMN inter-network FC of these altered networks, the decreased connectivity in PD OFF (compared to HC) increased in PD ON for all four networks without reaching the FC strength of HC.

There was a significant negative correlation of disease duration with connectivity between SMN and the motor/prefrontal network (IC7) in PD OFF. For PD ON, disease duration showed a significant negative correlation with SMN connectivity with the dorsal visual/superior parietal network (IC9), the right fronto-parietal network (IC4), a language associated network (IC15), and a component consisting of the salience, basal ganglia, and auditory networks (IC3). We found significant negative correlation for UPDRS-III improvement with SMN connectivity with the right fronto-parietal network (IC4) and for UPDRS-III score determined in medical OFF with SMN connectivity with the motor/prefrontal network (IC7) for patients in medical ON. There were no further significant correlations with SMN inter-network connectivity.

## Discussion

The current study investigates sensorimotor FC changes related to PD in a sample of patients under long-term dopaminergic treatment using a network-based resting-state fMRI approach. Our results indicate that the SMN is affected by the disease showing alterations, both, within the network and in interaction with other large-scale connectivity networks. All connectivity changes within the SMN and across networks observed in the medical OFF condition of PD patients partially normalized in medical ON pointing to a direct alleviating effect of dopaminergic treatment on PD-related impairment of SMN network integrity.

### Pathophysiological basis of sensorimotor network disruption in Parkinson’s disease

Dysfunction of sensorimotor regions is typically ascribed to denervation along the cortico-striatal pathway following nigrostriatal dopaminergic loss [38, 39]. Among the subparts of the striatum, the posterior putamen is predominantly connected to sensorimotor regions [40] and is most strongly affected by dopamine depletion in the disease [41, 42]. Indeed, several neuroimaging studies revealed reduced FC between the posterior putamen and sensorimotor areas [38, 43, 44]. Interestingly, likewise to our results, Helmich et al. primarily found sensory integration areas like the IPL and the parietal operculum as well as primary somatosensory areas affected by striatal decoupling, and not areas directly linked to motor function [38]. However, we primarily observed SMN connectivity alterations to other cortical networks rather than to the basal ganglia, which was likewise found in a previous work of Gratton et al. [45]. Hence, additionally to cortico-striatal denervation, alternative explanations for SMN disruption like more complex influences of neurotransmitter deficiency [46], e.g., secondary to fronto-striatal decoupling [47], network degeneration secondary to motor symptoms in terms of a reduced use, or effects of proteinopathy, may apply. These effects on cortico-cortical connections may possibly exceed the cortico-striatal alterations in advanced stages of the disease. On the other hand, possible cortico-striatal effects might be overlooked due to the mixed component that includes but is not restricted to the basal ganglia in our analysis (IC3).

Our findings highlight the importance of impaired network connections in the pathophysiology of PD. Within- and across-network alterations revealed by our study indicate that the disease clearly manifests and advances on a network basis and affects, both, the short-range and long-range connections of cortical sensorimotor areas. Overall, the changes observed between the SMN and other intrinsic connectivity networks are more pronounced than the within-network connectivity changes in our study. This is in line with previous studies investigating within-network as well as network-to-network connections in PD [45]. Impairment of inter-network connections as observed by the current study and various previous publications [45, 48–50] indicates that dysfunction in PD is in large part caused by impaired integration between brain networks and underlines the idea of understanding PD as a disconnection syndrome [51].

### Impaired sensorimotor integration in Parkinson’s disease

The SMN comprises the primary sensorimotor cortex as well as areas involved in motor task preparation, such as the premotor cortex and the SMA, and is activated in tasks of voluntary movements [10, 11]. Decoupling within the network as well as decreased inter-network FC between the SMN and the two motor-related networks revealed by our study indicates a disruption of the motor system in PD. However, a direct link to motor symptoms was only found in the negative correlation between UPDRS-III score and inter-network connectivity between SMN and the motor/prefrontal network for the medical ON resting-state scans in our study. Furthermore, the FC between SMN and this motor/prefrontal network showed negative correlation with disease duration, meaning that decoupling between these motor system networks advances with the progression of the disease. The fact that we did not observe more pronounced correlations of UPDRS-III with functional connectivity within SMN and across networks might indicate that network disruptions of the SMN could be present independently from motor symptom severity in advanced stages of the disease. On the other hand, it is conceivable that diverging pathological effects exist throughout different stages of the disease and across different motor subtypes, which lead to similar motor severity but different FC alterations of the SMN. In this case, more pronounced correlation effects could be obscured in the rather heterogeneous patient sample of our study. The observation that the correlation between UPDRS-III and inter-network connectivities is only found in the medical ON but not in the OFF state is quite peculiar. One possible explanation could be some kind of medical wear-off, in the sense that the alleviating effect of levodopa on inter-network FC diminishes with increasing motor severity, while the decreased functional connectivity across these networks per se might possibly be a more general pathological effect of the disease and less dependent on motor severity.

The main targets of within-network connectivity alterations found by our study are the left IPL and primary somatosensory cortex showing significant group differences. The IPL is implicated in several neurocognitive functions such as motor control, particularly of visually guided movements, (visuo-) spatial and non-spatial attention [52–55]. The most rostral part of the IPL, i.e., area PFt, which was found to be decoupled from SMN by the current analysis, is involved in action observation and imitation and is associated with the mirror neuron system [56, 57]. A recent connectivity-based parcellation and meta-analysis of the left IPL showed that this IPL sub-region is indeed most strongly connected to sensorimotor regions and involved in somesthesis, action execution, and motor/sensorimotor monitoring tasks [58]. Generally, it is conceived that the IPL integrates inputs from different sensory modalities, in particular visual, somesthetic, or proprioceptive, with motor signals to facilitate sensory guidance of movements and motor planning based on the perception of the own body and space [54]. Therefore, disconnection of the left IPL and the primary somatosensory cortex from the SMN observed in our study is probably related to impaired sensorimotor integration in PD. Indeed, PD patients expose a wide range of perceptual deficits and impairments of integrating sensory inputs for motor function [59]. It could be shown that deficits in proprioceptive- and visual-motor integration in PD patients in medical OFF significantly improve, when patients are under dopaminergic therapy, approaching the accuracy of HC [60]. Considering the integrative function of the IPL and the function of the SMN per se, it is very likely that the observed alterations in SMN connectivity are the neurobiological basis for these deficits in sensorimotor integration in PD. In this regard, the connectivity restoration of SMN decoupling by dopaminergic treatment observed in our study might reflect the dopamine-induced behavioral improvement of sensorimotor integrative function. Interestingly, the within-network FC in left IPL and left postcentral gyrus in PD ON even slightly exceeded the level of HC in our study. This overshooting of FC could be explained by enhanced recruitment of these areas to compensate for sensorimotor integration deficits induced by other areas or, alternatively, by some kind of local overdose effect of levodopa.

Area OP1 of the parietal operculum represents the secondary somatosensory cortex in humans and also facilitates somatosensory integration for motor function [33, 61]. The positive correlation between disease duration and FC within the SMN at area OP1, meaning that connectivity in this area increases with progressing disease, may be attributed to a compensation effect for the disrupted primary somatosensory and sensory integration areas.

Beside the two motor-related networks, across-network connectivity analysis revealed disconnections of the SMN from networks of the visual system, i.e., from an early visual network and a dorsal visual/superior parietal network. The first network comprises visual areas of the ventral and dorsal visual stream [62], where the ventral areas subserve extraction of basic features of the visual field like object shape, color, or motion in the posterior areas, and object, face, and word recognition in the more rostral areas [34, 35, 63, 64]. The dorsal visual/superior parietal network comprises occipital areas of the dorsal visual stream and areas of the posterior superior parietal lobule, which are mainly involved in higher level visuo-spatial processing, visual attention, and also visuo-motor integration [52, 65, 66]. Although PD is associated with a broad range of visual symptoms [67], which might be associated with the observed connectivity changes of these networks, it is likely that the inter-network disconnection between the SMN and the visual networks is also primarily related to the aforementioned impaired visuo-motor integration in PD. In this regard, our results highlight that disruption of sensorimotor integrative function of the SMN is not only driven by changes within the network, but particularly also by large-scale network-to-network disconnections.

### Sensorimotor network alterations in Parkinson’s disease

Our results are consistent with existing literature, which in the majority show decreased FC of the SMN. Using a graph-theoretical approach on resting-state FC data of non-demented PD patients, Gratton et al. showed that among all tested networks the SMN showed the greatest alterations between PD patients and controls [45]. Likewise to our results, they found decreased FC within the SMN and between the SMN and sensory networks, in particular visual networks. Interestingly, they also found stronger inter-network than within-network effects for the SMN, especially in regard to correlation with clinical/behavioral data. Esposito et al. investigated the within-network connectivity changes of the SMN in drug-naïve PD patients [68]. They found a diminished connectivity in PD patients in the SMA, which responded with a connectivity increase after a levodopa challenge administration. Likewise, Wu et al. demonstrated a decreased FC within the SMA in PD patients OFF medication, which normalized after levodopa administration [14, 69]. The reported PD-related connectivity decreases within the SMN normalizing with dopaminergic treatment are in line with the results of the current study. However, we did not observe connectivity alterations in the SMA, but in the left IPL and somatosensory cortex. These differences might be mainly attributable not only to the different patient collectives, i.e., advanced PD patients receiving dopaminergic medication for several years in our study compared to drug-naïve patients in Esposito et al. and a significantly lower disease duration in Wu et al., but also to different methodologies used for resting-state analyses between the studies. Furthermore, the SMN component from our ICA comprised only a rather small SMA representation, which could also explain why small effects in this area might have been missed. In a later study, Wu et al. also found disrupted FC in sensorimotor regions using a seed-based analysis of the pre-SMA and primary motor cortex [70]. Especially the reported decreased connectivity between pre-SMA and left IPL is consistent with our results of the within-network decoupling of the left IPL from SMN. In a sample of PD patients with freezing of gate and using an ICA-based approach comparable to our study, Canu et al. revealed even more pronounced FC decreases within the SMN compared to our study, but in different areas [71]. These differences in the manifestation of SMN decoupling are probably caused by the more specifically selected and principally more affected patient group in that study.

## Limitations

The current study entails some limitations. First, the included sample of PD patients is rather heterogeneous in regard to disease stage, duration, and symptom severity. It would be conceivable that functional connectivity changes of the SMN vary across the course of the disease or show distinctions between different motor types. However, we would argue that the heterogeneity in our sample increases the variability of connectivity alterations across the whole spectrum of the disease within the patient group and thereby should make the observability of significant effects less probable. This makes the observed changes in our study more robust and generalizable. Nevertheless, the current results may provide a substantial basis for further investigations on more selected samples of PD patients and to reproduce the current findings in different populations.

Likewise, medication schemes varied across included patients, as each patient received an individual dopaminergic medication plan resulting from individual optimization of treatment. Again, in our eyes, this diversity in medication protocols increases variability across the sample and makes the observed connectivity alterations and dopaminergic effects more robust and independent of specific drugs. In order to make antiparkinsonian treatment plans comparable, we calculated the LED according to Tomlinson et al. [16] and used it for correlation analyses with within- and across-SMN connectivity. Here, we did not find a significant correlation.

We examined PD patients in a medication OFF phase at least 12 h after withdrawal of all dopaminergic drugs. This approach is quite common to investigate patients under long-term medical treatment in a “drug-free” condition. However, we cannot exclude that residual medication effects from dopamine agonists or long-acting forms of dopamine remain after 12 h. Furthermore, FC alterations seen in medical OFF and medical ON may possibly be not attributable to disease-specific effects alone, but could in part be induced by long-term effects of dopaminergic treatment.

Patients were not systematically counterbalanced regarding the sequence of their scans, i.e., if they first underwent MRI examination in medical ON or OFF. We would not assume that the order of scans affects functional connectivity, but cannot rule out a sequence bias effect.

The choice to split the fMRI signal into 20 independent components in ICA is quite arbitrary. We applied elaborate preprocessing of the fMRI data including an ICA-based signal denoising method (FSL FIX), which makes the resulting preprocessed fMRI signal very clean from residual noise. Parcellations resulting from group-level ICA on such data almost exclusively entail components with neuronal signal and no noise components [20, 72], and the use of 15 to 30 independent components is quite common in such a setting. Additionally, in a recent machine learning-based classification approach between PD and HC, we could show that models using parcellations of 25 independent components perform better than models using 15, 50, 100, or 200 components, which might give a hint to the neurobiological most meaningful granularity to be within this range [73]. Twenty components were the best match to isolate most of the established intrinsic connectivity networks as single components and not splitting them into several sub-networks or having them merged into a single component. Especially for the SMN, choosing a higher dimensionality led to splitting the network into two or more components.

## Conclusion

In summary, PD is associated with extensive network changes of the SMN affecting its short- and long-range connections. These decouplings are probably associated with impaired sensorimotor integration in the disease. Several inter-network changes of the SMN are progressing with disease duration but seem rather independent of motor severity. Dopaminergic therapy can partially restore functional connections within the SMN and across networks, which may probably contribute to alleviation of neurological symptoms.

## Supplementary information

ESM 1 (PNG 600 kb)

High Resolution (TIF 1441 kb)
