# Supplementary figures and images for: Within- and across-network alterations of the sensorimotor network in Parkinson’s disease
Source: Neuroradiology. 2021 May 21;63(12):2073–85. doi: 10.1007/s00234-021-02731-w (PMC8589810; doi:10.1007/s00234-021-02731-w)

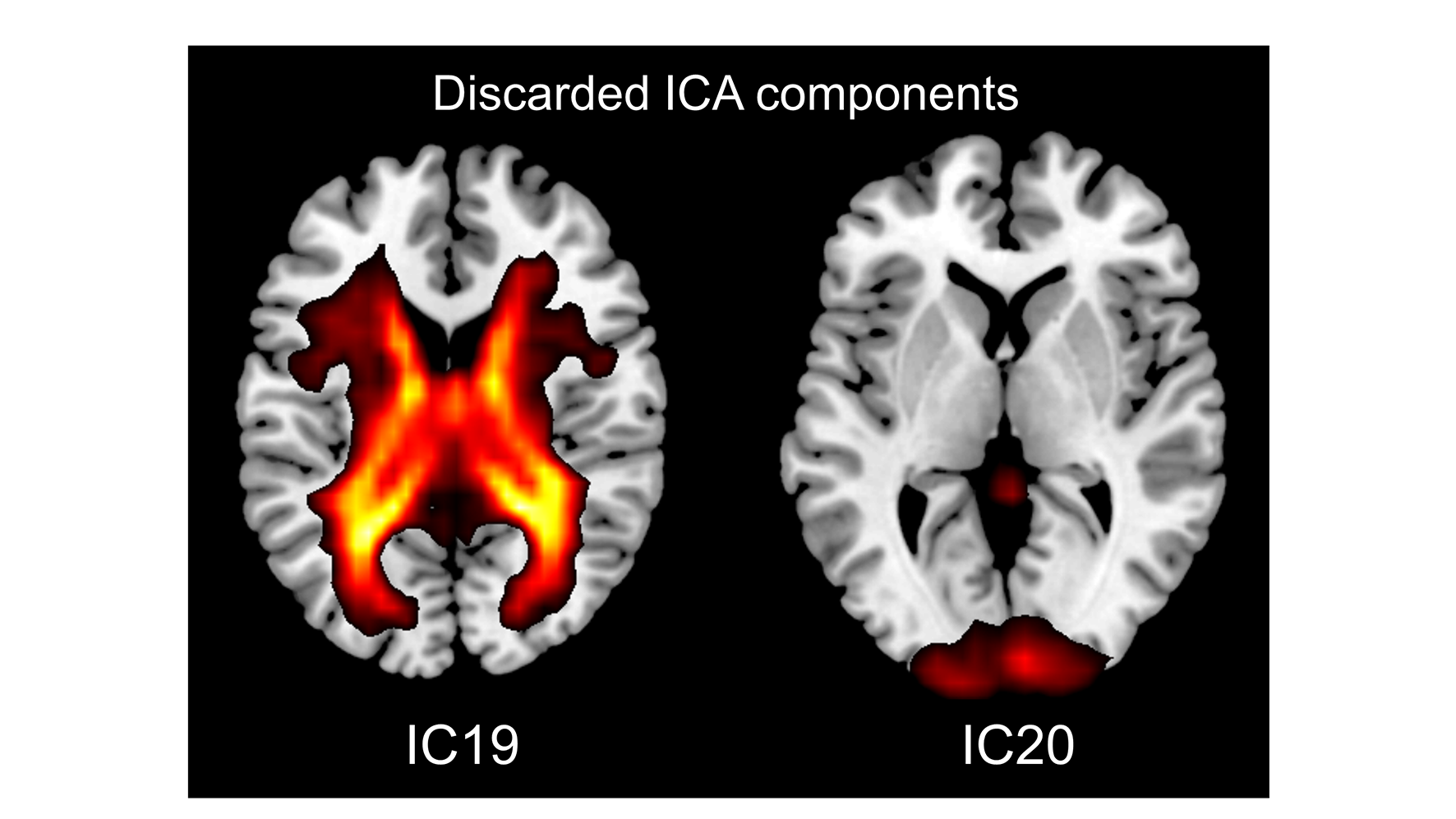

Supplement: Supplementary file 1 — (PNG 600 kb) [file 234_2021_2731_Fig1_ESM.png]

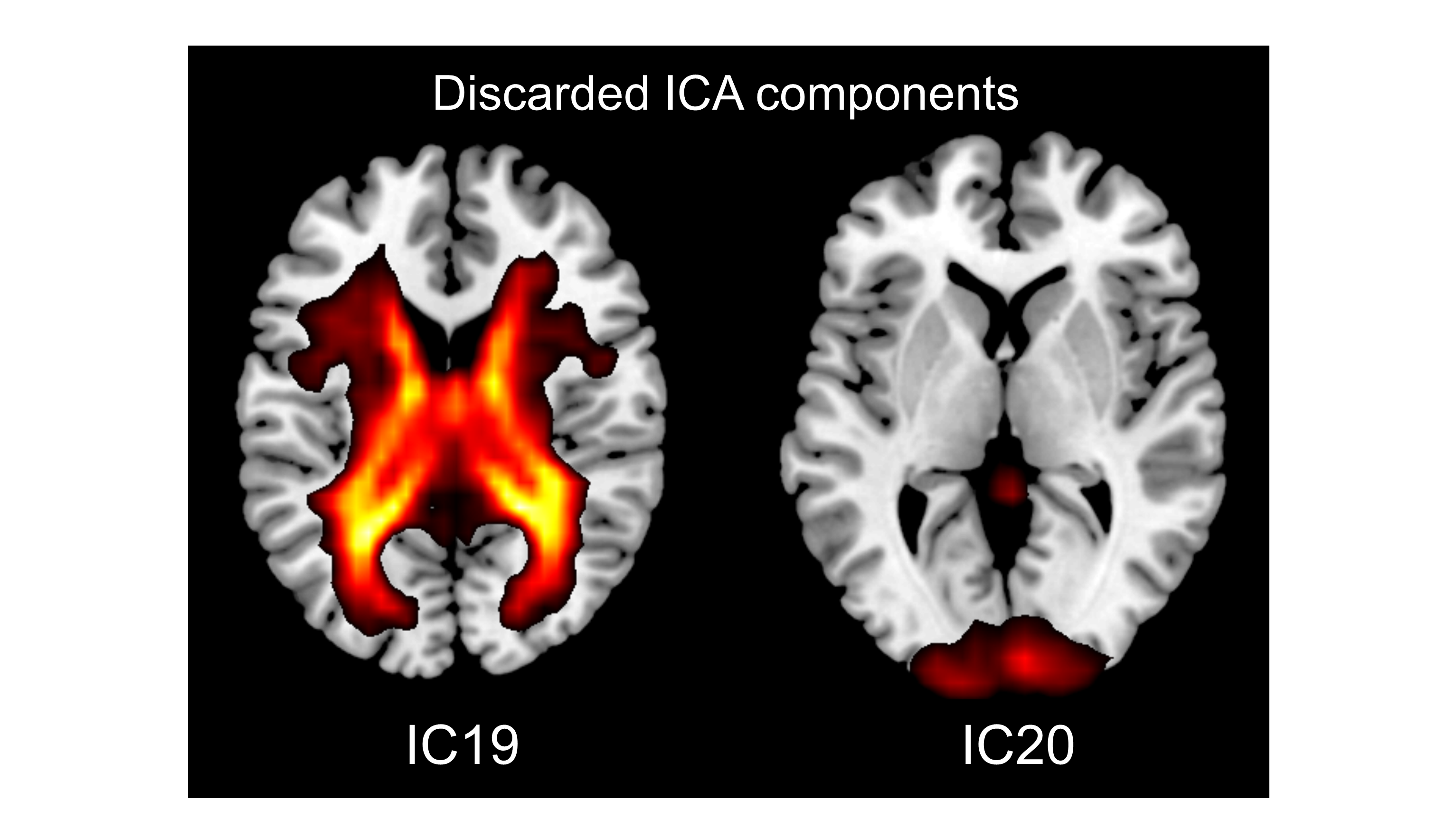

Supplement: Supplementary file 2 — High Resolution (TIF 1441 kb) [file 234_2021_2731_MOESM1_ESM.tif]
